# Instruments For Assessment Of Excessive Daytime Sleepiness In Brazilian Children And Adolescents: Systematic Review

**DOI:** 10.1590/1984-0462/2022/40/2020230

**Published:** 2022-04-04

**Authors:** Andrei Eduardo Bajerski, Incare Correa de Jesus, Francisco José de Menezes-Junior, Neiva Leite

**Affiliations:** a Universidade Federal do Paraná, Curitiba, PR, Brazil.

**Keywords:** Sleepiness, Disorders of excessive somnolence, Adolescent health, Adolescents, Children, Sonolência, Distúrbios do sono por sonolência excessiva, Saúde do adolescente, Adolescentes, Crianças

## Abstract

**Objective:**

To systematically review the literature on the instruments used to assess excessive daytime sleepiness (EDS) in Brazilian children and adolescents.

**Data source:**

A systematic review of the literature was performed in the databases MEDLINE PubMed, Scopus, Web of Science, LILACS, Scielo and SPORTDiscus, with no time limit for searches. The eligibility criteria were studies published in English and Portuguese, original articles that used questionnaires to assess EDS and whose sample consisted of Brazilian children and/or adolescents. As search strategy, the following terms were combined with Boolean operators “OR” and/or “AND”: drowsiness, disorders of excessive somnolence, excessive daytime sleepiness, day sleepiness, midday sleepiness, daytime sleepiness, adolescents, Brazil, Brazilian adolescents, and children.

**Data synthesis:**

Sixteen articles were selected, in which nine different instruments were applied to 8.240 children and adolescents from the South, Southeast, Midwest and Northeast regions of Brazil. The mean of methodological quality of studies was 16.1±1.9 points. The instruments most frequently used were the Pediatric Daytime Sleepiness Scale (PDSS) and Karolinska Sleepiness Scale (KSS), but only PDSS was shown reliable to assess EDS in Brazilian children and adolescents.

**Conclusions:**

The PDSS was the only instrument considered reliable to assess EDS in Brazilian children and adolescents. Further research on EDS in children and adolescents are suggested to perform the validation of other instruments for Brazil and present internal consistency values.

## Resumo

**Objetivo:**

Revisar sistematicamente a literatura sobre os instrumentos utilizados para avaliar a sonolência diurna excessiva (SDE) em crianças e adolescentes brasileiros.

**Fontes de dados:**

Revisão sistemática da literatura por meio das bases de dados eletrônicas Medical Literature Analysis and Retrieval System (MEDLINE), via PubMed, Scopus, Web of Science, Literatura Latino-Americana e do Caribe em Ciências da Saúde (LILACS), Biblioteca Eletrônica Científica Online (SciELO) e SPORTDiscus, sem limite temporal para as buscas. Os critérios de elegibilidade considerados foram estudos em inglês e português, artigos originais que utilizaram questionários para a avaliação da SDE e com amostra de crianças e/ou adolescentes brasileiros. Nas estratégias de busca, os seguintes termos foram combinados por meio dos operadores booleanos “OR” e/ou “AND”: drowsiness, disorders of excessive somnolence, excessive daytime sleepiness, day sleepiness, midday sleepiness, daytime sleepiness, adolescents, Brazil, Brazilian adolescents e children.

**Síntese dos dados:**

Foram selecionados 16 artigos, nos quais se identificaram nove instrumentos distintos aplicados em 8.240 crianças e adolescentes das regiões Sul, Sudeste, Centro-Oeste e Nordeste do Brasil. A média da qualidade metodológica dos estudos foi de 16,1±1,9 pontos. Os instrumentos utilizados com maior frequência foram o Pediatric Daytime Sleepiness Scale (PDSS) e o Karolinska Sleepiness Scale (KSS), porém somente o questionário PDSS apresentou valores de confiabilidade adequados para avaliar a SDE em crianças e adolescentes brasileiros.

**Conclusões:**

Atualmente, o PDSS foi o único instrumento que exibiu valores de confiabilidade para avaliar a SDE em crianças e adolescentes brasileiros. Sugere-se que pesquisas futuras relacionadas à SDE em crianças e adolescentes façam a validação no Brasil de outros instrumentos e apresentem os valores de consistência interna.

## INTRODUCTION

During adolescence, the maturation of sleep regulatory systems is associated with physiological factors and the influence of psychosocial and social situations that result in longer delays and shorter sleep time.^1^ Sleep is essential to repair wear and tear during waking hours. Therefore, it is a physical need essential for a healthy life.^2^ The difference between sleep duration measured in the laboratory^3^ and at home^4^ reinforces the interaction between bioregulatory mechanisms and psychosocial factors.^1^ Short sleep duration can bring consequences for many young people and is associated with cognitive deficits and poor health.^5^

Changes in the sleep cycle of adolescents can trigger problems that considerably influence an individual’s life,^2^ such as falling asleep on a bus or during school time, decreased school performance or even daytime alert, even leading to falling asleep while driving a car.^6^ In addition, school performance and productivity drop, while the incidence of psychiatric and health disorders increase.^7^ Excessive daytime sleepiness (EDS) is associated with decreased performance both at work and at school, being related to low learning and poor quality of life.^8^

Multiple sleep latency test (MSLT) and polysomnography are considered the gold standard methods for most sleep disorders and narcolepsy, respectively,^9^ but the application of both requires a laboratory and trained professionals, besides being expensive, which make its use in population studies unviable. In this setting, questionnaires to assess EDS are a valid, simple, practical, low-cost and viable option for studies with large populations.^10^

Although several self-report instruments have been developed and used by researchers, there is still no systematization of which instruments are the most common and adequate to measure EDS in Brazilian children and adolescents. Given the increasing use of electronic devices in all age groups,^11^ with the possibility of delaying the onset of sleep in children and adolescents, EDS can affect school performance and quality of life.^12^ Therefore, early diagnosis becomes important and represents an alert to improve the approach and learning of students.

This review allows to compare instruments for the analysis of the EDS available in the literature, and helps to identify the questionnaires that are most reliable and whose validity parameters are available in the literature. Therefore, the aim of this study was to to perform a systematic literature review about the instruments used to assess EDS in Brazilian children and adolescents.

## METHOD

This work was conducted based on the recommendations of the Preferred Reporting Items for Systematic Review and Meta-analyses: the PRISMA Statement,^13^ between January and April 2020, and was added to the International Prospective Registry of Systematic Reviews (PROSPERO) database (CRD42020139481).

The steps of search, selection, analysis of articles, application of methodological quality parameters and data extraction were carried out independently by three researchers (A.E.B., F.J.M.J. and I.C.J.). In cases of disagreement, a fourth researcher (NL) was consulted to decide on the divergent points.

Six electronic databases were searched according to field of knowledge and scientific relevance worldwide: Medical Literature Analysis and Retrieval System (MEDLINE) via PubMed (US National Library of Medicine, National Institutes of Health), Scopus (database) bibliographic), Web of Science (global bibliographic database), Latin American and Caribbean Literature on Health Sciences (LILACS), Online Scientific Electronic Library (SciELO) and SPORTDiscus (bibliographic database related to physical activity and exercise).

The search strategies were defined after the identification and selection of search descriptors, based on the Health Sciences Descriptors (DeCS), from the Latin American and Caribbean Center for Health Sciences Information (BIREME), and Medical Subject Headings (MESH), a controlled vocabulary used for indexing articles to PubMed. Thus, the following keywords were chosen in English and Portuguese: drowsiness, disorders of excessive somnolence, excessive daytime sleepiness, day sleepiness, midday sleepiness, daytime sleepiness, adolescents, Brazil, Brazilian adolescents and children. The keywords were combined using the Boolean terms “OR” and/or “AND”. No time delimitation was used in the searches or any other types of filters. References from selected studies were also searched for possible eligible works.

After applying descriptors, the studies identified in duplicity were removed, and then the following inclusion criteria were used:

- Studies that assessed daytime sleepiness using a self-report questionnaire.
- Studies conducted with humans.
- Original articles.
- Studies conducted with Brazilian children and/or adolescents.

After this phase, the following exclusion criteria were applied:

- Duplicate studies.
- Sample composed of adults and seniors.
- Non-Brazilian populations.
- Children and adolescents presenting psychological disorders.
- Samples that used medications that can interfere with sleep.

From the selected articles, we extracted data on the sample characteristics, such as age, biological sex, number of subjects, analysis of instruments identified with structural elements, reliability parameters, Cronbach’s alpha^14^ and validity. In cases of insufficient reporting of data related to the purpose of the study, the authors of the chosen articles were contacted.

The level of evidence and risk of bias of the selected studies were assessed using the Downs and Black checklist, to verify the quality and reliability of the results.^15^ This 27-item checklist assesses the quality, external validity, internal validity and power of the report. The maximum score a study can receive is 32, and the higher the score, the better the quality.

## RESULTS

**Figure 1.**
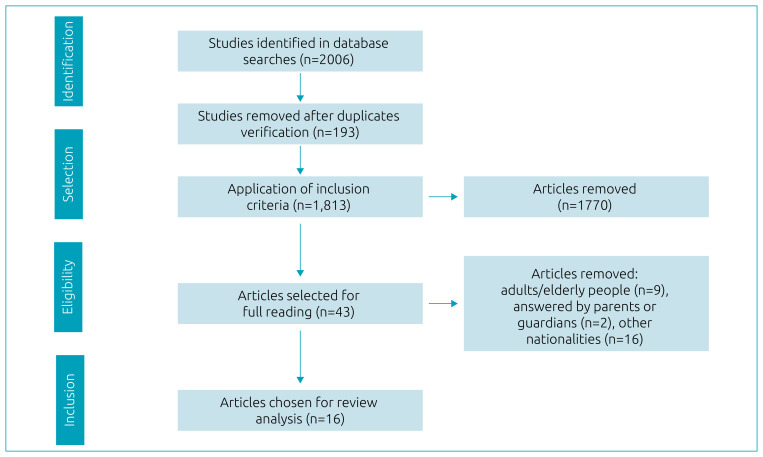
Flowchart of the process of selection of articles to compose the review.

After the search, 2,006 articles were identified with the combination of descriptors adopted. Of them, 193 were excluded after verification of duplicity, and 1,770 for not meeting the proposed criteria. Thus, 43 complete articles were selected and assessed for eligibility through full reading. At the end of this stage, nine studies were removed, as they had been carried out with adults and/or elderly people; two, because the instruments used reports by parents or guardians; and 16, for being of other nationalities. Finally, 16 articles were selected, in which nine different instruments were applied to 8,240 children and adolescents. The study selection and eligibility process is detailed in Figure 1.

Selection included studies with subjects aged between 7 and 19 years old, with samples of children and adolescents from the following Brazilian regions: Midwest (Souza et al.),^16^ Northeast (Almondes et al.)^17^ Southeast (Andrade et al.,^18^ Boscolo et al.,^19^ Fischer et al.,^20^ Vilela et al.^21^ and Del Ciampo et al.,)^22^ and South (Beijamini et al.,^23^ Beijamini et al.,^24^ Felden et al.,^25^ Felden et al.,^26^ Felden et al.,^27^ Felden et al.,^28^ Meyer et al.,^29^ Felden et al.^30^ and Ferrari Junior et al.^31^). There were also studies with stratification of sample in public and private schools (Boscolo et al.^19^ and Vilela et al.)^21^ and from capitals/municipalities in metropolitan regions and inland states (Felden et al.).^30^ The description of the selected studies is shown in Table 1.

**Table 1.** Description of selected studies.

Author	Location	Sample (n), sex	Age group (years) (mean±SD)	Instrument
Andrade et al.^18^	São Paulo, SP	66, ♀♂	13.5	Sleep questionnaire
Boscolo et al.^19^	Santo André, SP	45, ♀♂	Public school 13.4±0.6. Private school 13.1±0.5 e 13.3±0.5	Sleep questionnaire
Souza et al.^16^	Campo Grande, MS	378, ♀♂	16.9	ESS
Beijamini et al.^23^	Curitiba, PR	34, ♀♂	13.7±0.8	KSS
Beijamini et al.^24^	Curitiba, PR	21, ♀♂	13 and 14	KSS
Felden et al.^25^	Santa Maria, RS	1,126, ♀♂	16.2±1.3	Sleep habits questionnaire
Fischer et al.^20^	São Paulo, SP	40, ♀♂	15.8±0.8	KSS
Felden et al.^27^	Maravilha, SC	516, ♀♂	14.5±1.7	PDSS
Felden et al.^26^	Florianópolis, SC and Curitiba, PR	90, ♀♂	15.7±5.7	PDSS
Vilela et al.^21^	São Paulo, SP	531, ♀♂	Public school 13.2±2.2 Private school 14.4±1.9	CASQ
Del Ciampo et al.^22^	Ribeirão Preto, SP	535, ♀♂	10 to 19	Sleep habits questionnaire
Felden et al.^28^	São José, SC	1,132, ♀♂	14 to 19	PDSS
Meyer et al.^29^	São José, SC	1,132, ♀♂	16.1±1.1	PDSS
Almondes et al.^17^	Natal, RN	176, ♀♂	7 to 9	SDSC
Ferrari Junior et al.^31^	Paranaguá, PR	773, ♀♂	16.2±1.1	PDSS
Felden et al.^30^	Maravilha and São José, SC	1,645, ♀♂	Countryside 14.6±1.7 Metropolitan region 16.2±1.1	PDSS

♀: female; ♂: male; SD: standard deviation; ESS: Epworth Sleepiness Scale; KSS: Karolinska Sleepiness Scale; PDSS: Pediatric Daytime Sleepiness Scale; CASQ: Cleveland Adolescent Sleepiness Questionnaire; SDSC: Sleep Disturbance Scale for Children.

The analysis of quality and risk of bias of selected studies is shown in Table 2. The mean quality of the studies was moderate (mean score 16.1±1.9; range from 12 to 20). For the analysis of risk of bias, percentages were used as follows:

- <50%: high risk of bias.
- 50–75%: moderate risk of bias.
- >75%: low risk of bias.

**Table 2.** Quality and risk of bias of selected studies.

Scores from Downs & Black’s Checklist							
Studies	Instruments	Reporting	External validity	Bias	Confounding	Power	Total
		1–10	11–13	14–20	21–26	27	
Andrade et al.^18^	Sleep questionnaire	6 1	1	5	0 0	0	12
Boscolo et al.^19^	Sleep questionnaire	7 1	2	3	0 0	0	12
Souza et al.^16^	ESS	7	2	4	2	0	15
Beijamini et al.^23^	KSS	7	2	5	2	0	16
Beijamini et al.^24^	KSS	8	3	4	4	0	19
Felden et al.^25^	Sleep habits questionnaire	6	3	4	3	0	16
Fischer et al.^20^	KSS	7	3	3	4	1	18
Felden et al.^27^	PDSS	8	3	3	0	0	14
Felden et al.^26^	PDSS	6	3	3	4	0	16
Vilela et al.^21^	CASQ	8	1	4	3	0	16
Del Ciampo et al.^22^	Sleep habits questionnaire	6	2	3	3	0	14
Felden et al.^28^	PDSS	8	3	4	5	0	20
Meyer et al.^29^	PDSS	7	3	4	3	0	17
Almondes et al.^17^	SDSC	7	3	3	4	0	17
Ferrari Junior et al.^31^	PDSS	7	3	4	3	0	17
Felden et al.^30^	PDSS	7	3	4	5	0	19

ESS: Epworth Sleepiness Scale; KSS: Karolinska Sleepiness Scale; PDSS: Pediatric Daytime Sleepiness Scale; CASQ: Cleveland Adolescent Sleepiness Questionnaire; SDSC: Sleep Disturbance Scale for Children.

Thus, none of the studies presented a high risk of bias, five articles had moderate quality and 11 showed low risk of bias. The most common limitations identified as possible biases were related to the distribution and identification of main confounding factors in each group of individuals, the blinding of outcome measurers, and the detection of a clinically important effect when the p value (probability value) for a difference due to chance was less than 5%.

Among the instruments identified, the Pediatric Daytime Sleepiness Scale (PDSS)^26–30^ and the Karolinska Sleepiness Scale (KSS) were the most used ones.^20,23,24^ In addition to them, two different Sleep Questionnaires were also identified,^18,19^ along with the Epworth Sleepiness Scale (ESS),^16^ the Sleep Habits Questionnaire,^25^ the Cleveland Adolescent Sleepiness Questionnaire (CASQ),^21^ the Sleep Habits Questionnaire^22^ and the Sleep Disturbance Scale for Children (SDSC).^17^

The studies by Andrade et al.,^18^ Boscolo et al.,^19^ Souza et al.,^16^ Beijamini et al.,^23^ Beijamini et al.,^24^ Felden et al.,^25^ Fischer et al.,^20^ Felden et al.,^27^ Vilela et al.,^21^ Del Ciampo et al.,^22^ Felden et al.,^28^ Meyer et al.,^29^ Almondes et al.^17^ and Felden et al.^30^ did not present reliability criteria (Cronbach),^14^ while the studies by Felden et al.^26^ and Ferrari Junior et al.^31^ pointed out the alpha value.

The Cronbach’s alpha coefficient^14^ was presented for PDSS in the studies by Felden et al.^26^ (coefficient value = 0.784) and by Ferrari Junior et al.^31^ (coefficient value = 0.737), however no studies confirmed the reliability or the validity of KSS, Sleep Questionnaires, ESS, Sleep Habits Questionnaire, CASQ, SDSC and the Sleep Habits Questionnaire for Brazilian Children and Adolescents.

Table 3 shows the structuring components of each instrument assessing SDE. The PDSS has eight questions with answers arranged on a 4-point likert scale, from “never” to “always”. The scale score ranges from 0 to 32 points. Scores are analyzed continuously, and higher values represent more EDS. The KSS was structured according to nine points:

- 1Z: very alert.
- 3Z: alert.
- 5Z: neither alert nor sleepy.
- 7Z: sleepy (but does not fight sleep).
- 9Z: very sleepy (fights sleep).

**Table 3.** Questionnaires assessing daytime sleepiness.

	Instruments	Structure
1	ESS^16^	Eight items that estimate the trend of EDS in eight monotonous situations of daily life. The respondent must provide a score from 0 to 3, quantifying their tendency (probability) to fall asleep. Total points >10 represent increasing levels of SDE.
2	KSS^20,23,24^	Nine-point KSS: 1Z very alert, 3Z alert, 5Z neither alert nor sleepy, 7Z sleepy (but does not fight sleep), 9Z very sleepy (fights sleep).
3	PDSS^26–31^	Eight questions with answers arranged on a four-point Likert scale, from “never” to “always”. The scale score ranges from 0 to 32 points. Scores are analyzed continuously. The highest value represents more SDE. Cronbach’s alpha coefficient=0.78; and test-retest=0.72 (Brazilian children and adolescents).
4	Sleep Questionnaire^18^	The EDS question is presented in the following format: “Do you usually feel sleepy during the day, even without having slept late at night?”.
5	Sleep Questionnaire^19^	34 questions (adapted into 32 questions for this study) related to sleep pattern: subjective impression of the respondent regarding the quality of their sleep, sleep and wakefulness habits, and indicators of severity of the complaint or sleep problem presented.
6	Sleep habits questionnaire^25^	Sleepiness during school time was assessed by the question: “Considering the days you study, how often do you feel very sleepy in the classroom?”. Students could choose the alternatives: always, often, sometimes or never. Students who chose the alternatives “always” and “often” were classified as sleepy in the classroom.
7	CASQ^21^	Sixteen questions assessing the degree of sleepiness of adolescents, with limit values from 16 to 80 points, without a cutoff point for classifying the presence or absence of sleepiness.
8	SDSC^17^	Twenty-six questions divided into six groups of frequent sleep disorders in pediatrics (sleep onset and maintenance disorder, sleep-disordered breathing, awakening disorder, sleep-wake transition disorder, excessive sleepiness disorder and sleep hyperhidrosis). There are no cutoff points for classification, and values can vary between 26 and 130 in total.
9	Sleep habits questionnaire^22^	Previously validated questionnaire that allowed to determine some characteristics related to sleep during the period when the student is not at school, bedtime and waking up on weekdays and weekends, what the student does before bedtime (reads, uses the computer, plays games, uses telephone, listens to music), if they sleep during the day, if they take a long time to fall asleep, if they have daytime sleepiness, if their sleep is interrupted at night, if they wake up by themselves or need to be awakened. a) take a long time to fall asleep (more than 30 minutes); b) wake up very early (between 5 and 7 in the morning); c) sleep during the day (more than 30 minutes); d) wake up at night (not considering going to the bathroom).

EDS: excessive daytime sleepiness; ESS: Epworth Sleepiness Scale; KSS: Karolinska Sleepiness Scale; PDSS: Pediatric Daytime Sleepiness Scale; CASQ: Cleveland Adolescent Sleepiness Questionnaire; SDSC: Sleep Disturbance Scale for Children.

The ESS includes eight items that estimate the trend of EDS in eight monotonous situations of daily life. A score >10 represents increasing levels of EDS, and the EDS question is presented in the Sleep Questionnaire as follows: “Do you often feel sleepy during the day, even if you don’t do to sleep late at night?”

The Sleep Questionnaire has 34 questions (adapted into 32 questions) addressing sleep pattern. Through the sleep habits questionnaire, sleepiness in the classroom was assessed by the question: “Considering the days you study, how often do you feel very sleepy in the classroom?”, and students could choose between the alternatives: always, often, sometimes or never. Students who chose the alternatives “always” and “often” were classified as sleepy in the classroom.

The CASQ has 16 questions about the degree of drowsiness/sleepiness of adolescents, and its limit values are from 16 to 80 points, without a cutoff point for classifying the presence or absence of drowsiness. The SDSC includes 26 questions divided into six groups of frequent sleep disorders in the field of pediatrics. There are no cutoff points for classification, and values can vary between 26 and 130 in total. The sleeping habits questionnaire determines some characteristics related to sleep when the student is not at school.

The number of questions related to sleepiness in the questionnaires varies. Three questions assess sleepiness in the SDSC, while in PDSS, CASQ and ESS are fully dedicated to this matter. It is also important to point out that the age group for which each questionnaire was validated may differ.

## DISCUSSION

The aim of this study was to perform a systematic literature review on the instruments used to assess EDS in Brazilian children and adolescents. The main findings, among the 16 studies, indicate the use of nine research instruments to assess EDS in Brazilian children and adolescents, however two of them appeared more frequently: PDSS and KSS. Only the PDSS was evaluated for reliability and validity in the studies by Felden et al.^26^ and Ferrari Junior et al.,^31^ with a satisfactory Cronbach^14^ coefficient.

Thus, an important limitation in most studies was that few of them reported the validity and reliability of the instruments used. In this sense, only the PDSS was validated for the population of Brazilian children and adolescents. The PDSS was created by Drake et al.^32^ and is composed of eight multiple-choice questions, each having five response options on a likert scale, where higher scores, ranging from 0 to 32, indicate more sleepiness. Felden et al.^26^ verified the validity of PDSS with 90 children and adolescents from southern Brazil and identified satisfactory reliability, as values between 0.7 and 0.8 are generally acceptable for this analysis. This result suggests the validity of the questionnaire for the Brazilian infant-juvenile population.

The SDSC, developed by Bruni et al.,^33^ in divided in two sections. The first collects demographic, behavioral and clinical data, information about past illnesses and medical status with specific questions about disorders or disorders that can affect sleep. The second is composed of 26 items on a Likert-type scale, with values from 1 to 5, and with wording arranged so that higher numerical values reflect greater severity of symptoms. Ferreira et al.^34^ carried out the translation, cultural adaptation and validation of the questionnaire with 100 children and adolescents aged between 3 and 18 years, and living in the Southeast region of Brazil. According to the authors, the level of internal consistency was satisfactory, with values ≥0.55.

However, information about the application of these instruments is still limiting, since the PDSS was applied to children and adolescents from two private schools, one in Florianópolis (SC) and the other in Curitiba (PR); therefore, the young students of Brazilian public schools were not encompassed. The SDSC pointed out that parents or guardians participated in data collection, but the article does not specify whether children and adolescents were enrolled in public or private schools. In both articles, the maturational level and the levels of physical fitness or sedentary behavior were not evaluated, which could compromise the results.

According to the bias analysis, on average, the risk of bias of studies was classified as moderate and high. The most significant methodological flaws were:

- Distribution of the main confounding factors in each group of individuals to be compared clearly described.
- Main adverse effects that could result from the intervention reported.
- Characteristics of lost participants described.
- Blinding of participants to the type of intervention they received.
- Blinding of outcome measurers.
- Blind randomized intervention to patients and staff until recruitment was complete and irrevocable.
- Adequate adjustment of confounding factors in the analyses.
- Study with sufficient power to detect clinically important effects when the p-value (probability value) was less than 5%.

Meyer et al.^10^ systematically reviewed the use of PDSS in 26 studies after the inclusion criteria, and the following items were analyzed: population, sample, age group, location, score, design, mean and standard deviation of PDSS and results. However, the article did not indicate the use of Cronbach’s alpha^14^, which is fundamental for reliability.

In order to compare, when systematically reviewing the literature on the evidence associating sleep disorders and quality of life in children and adolescents with juvenile idiopathic arthritis, Carneiro et al.^35^ did not assess the quality of the articles, only pointing out that, despite all the identified questionnaires having been validated in the literature, there is a bias between the instrument and the respondent. The main questionnaire used in the studies included in the review was the Children’s Sleep Habits Questionnaire, which mainly assesses the clinical aspects of pain and functional capacity.

To our knowledge, this is the first study that systematically verified the main self-report instruments to assess EDS in Brazilian children and adolescents. During the searches for articles for this systematic review, including other reviews on the same topic, a considerable increase in research related to EDS in children and adolescents was noticed in recent years, but not all of them present the internal consistency of Cronbach’s alpha^14^ in their results.

In the study developed by Felden et al.,^26^ in which the PDSS was translated into Portuguese and validated for the Brazilian population, it presented satisfactory reliability results (Cronbach’s alpha=0.784), close to those found by Drake et al.^32^ (Cronbach’s alpha=0.81), the creators of the questionnaire. Similarly, Ferreira et al.^34^ carried out the translation, cultural adaptation and validation of the SDSC questionnaire with 100 children and adolescents aged between 3 and 18 years and living in Southeast of Brazil. This instrument was developed by Bruni et al.,^33^ and its level of internal consistency was ≥0.55.

The results of this study show that the instrument most indicated and used by researchers interested in aspects related to sleep, more specifically on EDS, is the PDSS. Even so, it is worth noting the lack of studies proposing cutoff points for the score obtained from the participants’ responses, so that they are widely accepted in the literature. Felden et al.^27^ analyzed the scores based on tertiles in multinomial analysis:

- First tertile: 0 to 12 points.
- Second tertile: 13 to 18 points.
- Third tertile: above 18 points.

Ludwig et al.^36^ used the cutoff of 15 points proposed by Meyer et al.^29^ in a sample of 365 students aged 4 to 12 years, their parents and their teachers at a large regional primary school in Queensland, Australia. When evaluating 1,132 adolescents aged between 14 and 19 years of both sexes and from a public school in the city of São José (SC), Meyer et al.^29^ proposed the aforementioned cutoff point for the definition of EDS for both biological sexes. Poor sleep quality was the reference most strongly related to daytime sleepiness.

In addition to the validation and reliability of the instruments to be used, factors such as the month of data collection, whether the participants are considering their routine to answer the questionnaire, the time when students would participate and the class in which they would respond to the questionnaires should be taken into account. It is, then, noteworthy that the motivation to be alert, the variation of daily routine, effects associated with the day of the week and features associated with sleep the night before can influence the effect of EDS on academic results and data collected in research.^36^

The importance of defining cutoff points for the scales for future studies is also highlighted, as to improve the understanding of results and analyses carried out, and to contribute to the discussion around findings. In parallel, studies evaluating daytime sleepiness and the factors that can cause EDS in young people with the presence or absence of a sleep disorder are suggested to be conducted with Brazilian children and adolescents from both public and private schools.

In conclusion, it appears that the PDSS was the only instrument that showed reliability values in studies with Brazilian children and adolescents, considering only questions answered by the young people themselves as having a satisfactory quality. There is an important limitation of studies that aimed to identify the validity of other instruments. Therefore, the PDSS is suggested as an appropriate instrument to assess EDS in Brazilian children and adolescents. Future studies are recommended, with the aim of testing the validity of other instruments, even the PDSS, comparing their results with the gold-standard method for diagnosis of EDS, based on the multiple sleep latency test and self-reported sleep questionnaires in populations with different economic, social and behavioral characteristics.
